# Preparation of Polyurethane Monolithic Resins and Modification with a Condensed Tannin-Yielding Self-Healing Property

**DOI:** 10.3390/polym11111890

**Published:** 2019-11-15

**Authors:** Jéssica Verger Nardeli, Cecílio Sadao Fugivara, Elaine Ruzgus Pereira Pinto, Wagner Luiz Polito, Younes Messaddeq, Sidney José Lima Ribeiro, Assis Vicente Benedetti

**Affiliations:** 1 Department of Physical-chemistry, Institute of Chemistry, São Paulo State University (UNESP), PO Box 355, Araraquara 14801-970, SP, Brazil; sadao.fugivara@unesp.br; 2 Department of General and Inorganic Chemistry, Institute of Chemistry, São Paulo State University (UNESP), PO Box 355, Araraquara 14801-970, SP, Brazil; elainerpp@gmail.com (E.R.P.P.); politowl@iqsc.usp.br (W.L.P.); younes.messaddeq@copl.ulaval.ca (Y.M.); sidney.jl.ribeiro@unesp.br (S.J.L.R.)

**Keywords:** condensed tannin, self-healing, polyurethane resins, condensed tannin-modified PU resin, crambe oil, castor oil

## Abstract

Resins of polyurethane were prepared from vegetable oils (crambe and castor) and modified by adding green corrosion inhibitor (condensed tannin). The oils were characterized by gas chromatography with flame-ionization detection (GC-FID), attenuated total reflectance Fourier transform infrared spectroscopy (FTIR-ATR) and thermogravimetric analysis (TGA). The reaction was monitored by characterizing the intermediate products (polyester and prepolymer). The polyester was characterized by solubility in methanol, acidity index, hydroxyl groups and FTIR-ATR, and the prepolymer was characterized by solid content, solvent content, isocyanate (NCO) groups and FTIR-ATR. The formation of PU resins was confirmed by FTIR-ATR and TGA, and the presence of tannin particles incorporated in the coating can be observed by optical microscopy (OM). The absence of the band attributed to NCO in FTIR-ATR spectra of the resins confirmed the complete reaction between polyester and prepolymer. The OM observation and a video demonstrate that Polyurethane (PU)-modified with condensed tannin resin presents self-healing effect, probably through the formation of new hydrogen bonds when in contact with deionized water. Therefore, these results open possibilities for new synthetic routes aiming at improving the very important self-healing property for protecting metals and their alloys against corrosion, extending significantly the metallic materials lifetime as previously demonstrated by our group.

## 1. Introduction

Modern life relies on polymers, from the materials that are used to make airplanes and cars to those with sophisticated applications in medicine. However, most polymers are derived from petrochemicals [1]. Polyurethane (PU) resin is one of the widely used materials with great potential for multipurpose applications due to its excellent physical, chemical, and mechanical properties [2,3,4,5,6,7,8]. Industrial processing of these compounds is still widely carried out by relying on the processing of petrochemical industries, where raw materials derived from natural gas and petroleum are limited and cannot be renewed [9]. It is important to remember that some petrochemical polymers are biodegradable, and not all bioderived polymers will be biodegraded [1]. Polyurethanes are also applied on metal surfaces forming barrier coatings to protect them against corrosion in aggressive environments [10,11].

**Figure 1:**
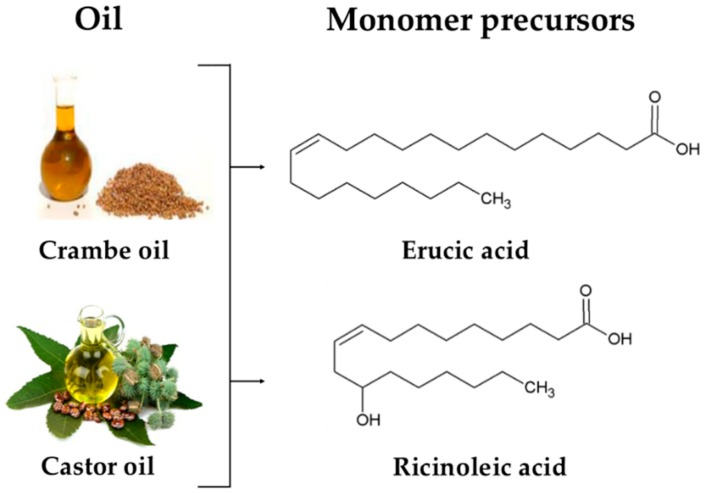
The main fatty acids present in oils.

In recent years there has been a growing interest in developing sustainable polymer coatings that reduce the use of petrochemical-based polymers [12]. Industrial and consumer interests in the development of green materials from abundant renewable resources have increased as they are readily available, of low cost, nontoxic and biodegradable [13]. Crambe oil from *Crambe abyssinica* plant fills these characteristics and is composed of different fatty acids, with specific properties, which are absent in other raw materials, being considered biocompatible and biodegradable [14,15]. *Crambe abyssinica* is a plant that can also provide a suitable raw material for higher-value products, such a, polymers, polymer additives, surfactants, personal care products and pharmaceuticals [16,17,18]. Castor oil is inherent biodegradable, of low cost, almost non-toxic, industrially [14,15] favorable, and easily available [19,20]. It is one of the most important renewable resources to produce polymers, including the preparation of polyurethanes [21], replacing petroleum-based products. Figure 1 illustrates the main fatty acids present in oils.

Crambe oil is the most important component of polyester, and castor oil of the prepolymer.

Why and how the resins here studied were drawn? The initial studies of crambe oil-based polyurethane indicated great dependence of the electrochemical response on the nature of the resin composition [22,23], and it was also observed that after applying a scratch on the coating surface, the self-healing property was present in a resin-based coating, in which the pristine was less resistant to corrosion [24]. Conversely, the pristine that showed better corrosion resistance did not present the self-healing property [24]. This has called our attention to investigate the corrosion resistance of different coatings formulations by changing the components and their proportions. Besides the base components (crambe and castor oils, and aliphatic diisocyanate), two other components of the coating are trimethylolpropane (TMP), a crosslinking agent [25,26], and phthalic anhydride (PhA) that takes part in the polyesterification and easily reacts with OH groups present in TMP and mono- or diglycerides. This reaction results in polyesters formation (flexible segment) [25,26]. Then, we choose, initially, to use two different quantities of the crosslinking agent (TMP) maintaining constant the mass of PhA, fabricating two different polyesters [24]. Then, the idea was to study the effect of the crosslinking agent on the corrosion resistant ability of the coating. The other part composing the coatings is the prepolymer, which is obtained by reacting hexamethylene diisocyanate (HDI, which contains bifunctional NCO terminal groups) with OH groups from different sources to produce the rigid segment. The final resin is obtained by reacting both flexible and rigid segments in a chosen polyester and prepolymer proportion.

**Figure 2:**
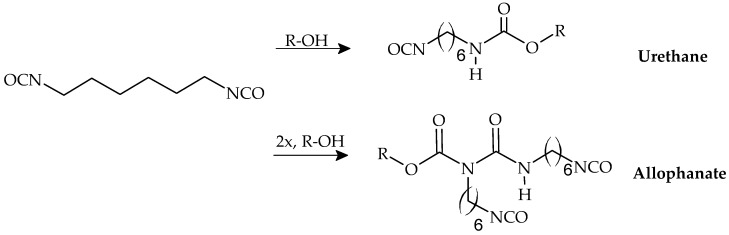
Main reactions of prepolymer in the curing process [30], modified by the authors.

HDI represents the most important class of polyisocyanates used today in the fabrication of polyurethane resins. Resins prepared with aliphatic diisocyanates provide excellent resistance to chemicals and to yellowing [27,28]. This is one of the main advantages of using aliphatic diisocyanates compared to aromatic ones, whose resins undergo oxidation easier, especially when exposed to UV light, than those prepared from aliphatic diisocyanates [29]. HDI can react with many chemicals [30] and in our system the main reactions of HDI to form the prepolymer are drawn in Figure 2. 

In order to increase the amount of hydroxyl groups in the final resin structure and develop a smart coating, condensed tannin was incorporated into the synthesis. 

Tannin in vascular plants occurs as two types, condensed and hydrolysable. Hydrolysable tannin is made up primarily of gallic acid while condensed tannin is made up from three-ring flavanols [31] and is largely used to produce adhesives, foams, biofoams [32] and many tannin-based polymers using different hardeners [33]. Tannins are natural polyphenolic antioxidants [34] and contain multiple adjacent hydroxyl groups and exhibit specific affinities for many metal ions [34]. Tannin, when added to the electrolyte or in the primer, protects metallic surfaces against corrosion [35,36,37,38,39,40,41], and when encapsulated tannin was added to sol–gel films of methyltrimethoxysilane and tetraethylorthosilicate, but when directly added to the sol–gel film was leaching [42]. However, when condensed tannin was directly incorporated to the crambe oil-derived polyurethane resin have conferred the self-healing property to the coating and tannin leaching was negligible [43], which attributed to this material excellent protection of AA2024 alloy against corrosion in chloride solutions. 

This work aims at formulating, preparing and characterizing PU resins unmodified [22,24] and modified with condensed tannin used as corrosion inhibitor of aluminum and aluminum alloys [43], and demonstrating the self-healing effect in the condensed-tannin-modified PU resin monoliths, which significantly increases the service life of corrosion-resistant coatings with artificial defects or damaged in service. The investigation of self-healing property in monoliths when compared to the coated substrate may give insight about the influence of the metallic substrate in the corrosion resistance performance of the coating.

## 2. Materials and Methods 

### 2.1. Materials

Crambe oil (CO) provided by Foundation Mato Grosso do Sul (Campo Grande, Brazil) [44], castor oil (CAO) provided by Asher^®^ (São Carlos, Brazil), condensed tannin species extracted from mimosa (*Acacia mearnsii*) [45,46] provided by TANAC^®^ (Montenegro, Brazil). Trimethylolpropane (TMP, CAS number 77-99-6), phthalic anhydride (PhA, CAS number 85-44-9), butylated hydroxyl toluene (BHT, CAS number 128-37-0), lithium hydroxide (LiOH, CAS number 1310-65-2), propylene glycol (PG, CAS number 57-556), n-butyl acetate (BA, CAS number 123-86-4), ethyl glycol acetate (EGA, CAS number 111-15-9), hexamethylene diisocyanate (HDI, CAS number 212-485-8), stannous octoate (SO, CAS number 301-10-0) were used to prepare the organic resin. All reagents and solvents are analytical grade and were purchased from Sigma-Aldrich^®^ (St. Louis, MO, USA).

### 2.2. Characterization Techniques

#### 2.2.1. Raw Materials

##### Quantification by Gas Chromatography with Flame-Ionization Detection (GC-FID)

**Scheme 1:**
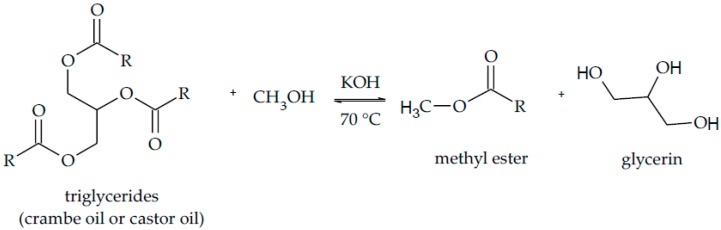
Transesterification of vegetable oils to form methyl esters.

For this analysis, both vegetable oils were transesterified to form methyl esters (Scheme 1). The reactions were carried out in a flask equipped with a reflux condenser, and the raw material (50 g) that remained under stirring and heating was introduced until reaching a temperature of 70 °C. In parallel, amounts of potassium hydroxide catalyst (KOH, 0.5g) and methanol (CH_3_OH, 91 mL) were added and homogenized. The reaction was kept under constant stirring at a temperature of (70 ± 5) °C for 3 h at reflux. At the end of the reactions, the product was separated from glycerol, excess methanol, catalyst and other soluble impurities in the polar environment, followed by washing of the methyl esters and subsequently analyzed/quantified by gas chromatograph equipped with a flame ionization detector (GC-FID). The methyl esters were dissolved in heptane and the analysis performed in a Shimadzu GC-2010 GC-FID (Kyoto, Japan) and a Zebron ZB -5HT column (30 m × 0.32 mm × 10 µm), helium (carrier gas with velocity 54.0 cm s^−1^), stationary phase 5% phenyl and 95% dimethyl polycyclohexanol No. 314134.

##### Attenuated Total Reflectance Fourier Transform Infrared Spectroscopy (FTIR-ATR)

Raw materials (crambe and castor oil), the intermediates (polyester, prepolymer) and final products (PU resin, and PU-modified resin) were analyzed by means of a FTIR-ATR using a Vertex70 Bruker spectrophotometer (Billerica, MA, USA) with an Attenuated Total Reflectance accessory (scans = 64, energy scanning from 590 to 4000 cm^−1^, resolution = 4 cm^−1^). 

#### 2.2.2. Polyester

##### Hydroxyl Groups

This analysis confirms whether the modification reaction of crambe oil was successful by determining the quantity of hydroxyl groups added to the chemical structure. The quantity of hydroxyl groups was determined in triplicate according to ASTM E222 [47] and calculated using Equation (1):

(1) $$I_{\mathrm{OH}}=\frac{V_{B}+\left(\frac{m_{\mathrm{sm}}\times V_{\mathrm{IAl}}}{m_{\mathrm{IAl}}}\right)-V_{\mathrm{KOH}}}{m_{\mathrm{sm}}}\times 56.1$$

where V_B_ = volume of alcoholic KOH standard solution necessary to titrate the blank (oil) (mL); V_KOH_ = volume of the alcoholic KOH standard solution necessary to titrate the sample (mL); V_IAl_ = volume of the standard solution of alcoholic KOH spent on I_Al_ (mL); m_sm_ = sample mass (g); m_IAl_ = sample mass (g) for the determination of index acid (I_AI_).

#### 2.2.3. Prepolymer

##### Solid Content and Solvent Content

The solid content was determined for the prepolymer and indicates the quantity of remaining reagent after the solvent curing and evaporation process [25,26]. For analysis, Petri dishes were used. Each Petri dish was weighed empty followed by the addition of an amount of prepolymer (~1 g). The samples were then kept at room temperature (25 ± 5) °C for 24 h. The calculation was performed according to Equations (2) and (3).

(2) $$\mathrm{Solid}\;\mathrm{content}\;\left(\%\right)=\frac{\mathrm{dry}\;\mathrm{prepolymer}\;\mathrm{mass}\;\left(g\right)}{\mathrm{mass}\;\mathrm{of}\;\mathrm{the}\;\mathrm{liquid}\;\mathrm{prepolymer}\;\left(g\right)}\times 100$$

(3) Solvent content (%)=100−(solid content)

##### NCO Groups

The quantity of NCO groups was determined in triplicate according to ASTM D2572 [48,49] and calculated according to Equation (4). The NCO groups defines the mass percentage of isocyanate groups that are available to react with polyester, water, etc.

(4) $$\%\mathrm{NCO}=\frac{\left[\left(B-V\right)\times N\times 0.0420\right]}{W}\times 100$$

where B = volume of HCl solution used to titrate the blank (without the sample) (mL); V = volume of HCl solution used to titrate the sample (mL); N = concentration of HCl solution; W = sample mass (g).

#### 2.2.4. PU Resin and PU-Modified Resin

PU resin and PU-modified resin were characterized by FTIR-ATR.

##### Raman Spectroscopy

All the samples were also analyzed by solid state Raman using a Fourier transform Raman spectrometer (Bruker RAMII model, Billerica, MA, USA). For each Raman spectrum, 200 scans were performed in the range of 4000–500 cm^−1^ with a resolution of 4 cm^−1^. The source of radiation was a leisure with a wavelength of 1064 nm and a power of 100 mW with acquisition time of 6 min.

##### Thermogravimetric Analysis (TGA)

The thermal stability of raw materials (vegetable oils) and resins (PU and PU-modified) was analyzed by thermogravimetric analyzer (TGA, Hitachi STA 7200, Tokyo, Japan), performed from 25 °C to 550 °C at 10 °C min^−1^ under nitrogen gas at a flow rate of 100 mL min^−1^.

##### Optical Microscopy (OM)

The surface of resins with an artificial scratch (size 1000 µm × 60 µm) using a scalpel blade was recorded during 4 h of immersion in a thin layer of deionized water using a system [50] that allows to monitor the surface with a stereomicroscope Carl ZEISS Jena (Oberkochen, Germany) coupled to a computer and TOP VIEW^®^ software (SPECwise, Inc., West Palm Beach, FL, USA).

## 3. Results and Discussion

### 3.1. Characterization of Raw Material

#### 3.1.1. Quantification by Gas Chromatography with Flame-Ionization Detection (GC-FID)

Results from GC-FIG of methyl esters (crambe and castor) are shown in Table 1. The results suggest that the major component for crambe methyl esters is derived mainly from erucic acid (13-*cis*-docosenoic acid), while for castor methyl esters, the largest component is ricinoleic acid (12-hydroxy-9-*cis* octadecenoic).

#### 3.1.2. Attenuated Total Reflectance Fourier Transform Infrared Spectroscopy (FTIR-ATR)

**Figure 3:**
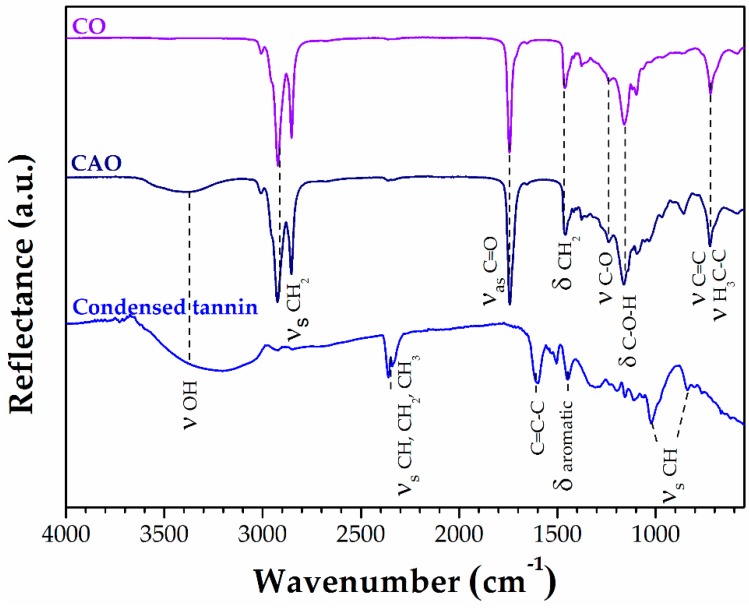
FTIR-ATR spectra for the crambe oil, CO (**—**), castor oil, CAO (**—**) and condensed tannin (**—**).

Figure 3 illustrates the FTIR-ATR spectrum for crambe oil, castor oil and tannin and the main attributions of the different bands in FTIR-ATR (normalized intensity of bands) stand out. 

The FTIR-ATR spectrum of crambe oil evidences the absence of –OH band, as expected because crambe oil has no hydroxyl in its structure; the spectrum for castor oil evidences the presence of –OH (3425 cm^−1^). In the case of condensed tannin, the absence of band C=O (~1700 cm^−1^) [40,43] confirms that the structure is a condensed (condensed tannin) [51] and contributes to the insertion of OH (~3340 cm^−1^) that favors the self-healing effect when the resin is applied to protect aluminum alloys [43]. 

#### 3.1.3. Thermogravimetric Analysis (TGA)

**Figure 4:**
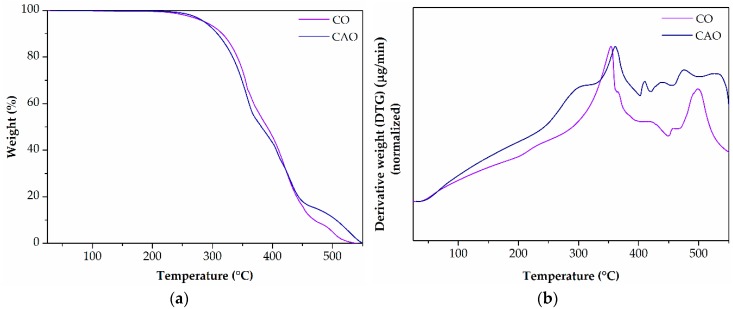
TGA weight loss (**a**) and derivatives of TGA data (**b**) of crambe oil, CO (**—**), castor oil, CAO (**—**).

Representative dynamic thermogravimetric analysis (TGA) results of crambe oil and castor oil are shown in Figure 4.

Crambe and castor oils exhibit no appreciable weight loss before 250 °C, indicating good thermal stability. The most stable raw material was crambe oil-CO (300 °C), followed by castor oil-CAO (294.4 °C). Both oils presented thermal decomposition in three consecutive stages between 245–535 °C (crambe oil-CO) and 240–555 °C (castor oil-CAO). These values indicate high purity of oils, with no presence of inorganic compounds. DTG curves present a series of exothermic events, which are attributed to the oxidative degradation of organic matter (oil structure).

### 3.2. Main Chemical Reactions for the Polyester and Prepolymer Preparation and Products Characterization

#### 3.2.1. Polyester Preparation

**Figure 5:**
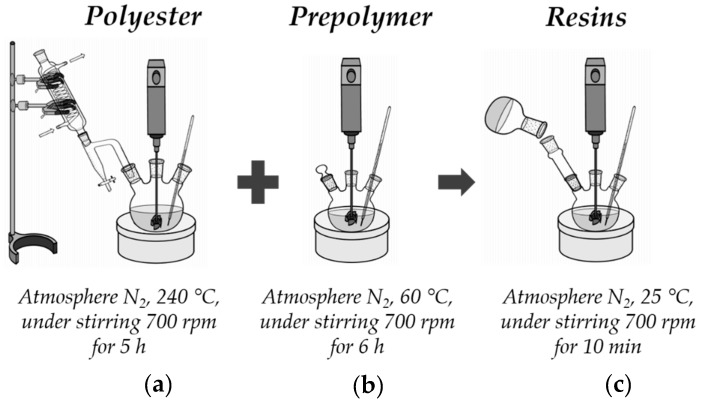
Reaction system for: (**a**) polyester, (**b**) prepolymer and (**c**) PU and PU-modified resins.

**Scheme 2:**
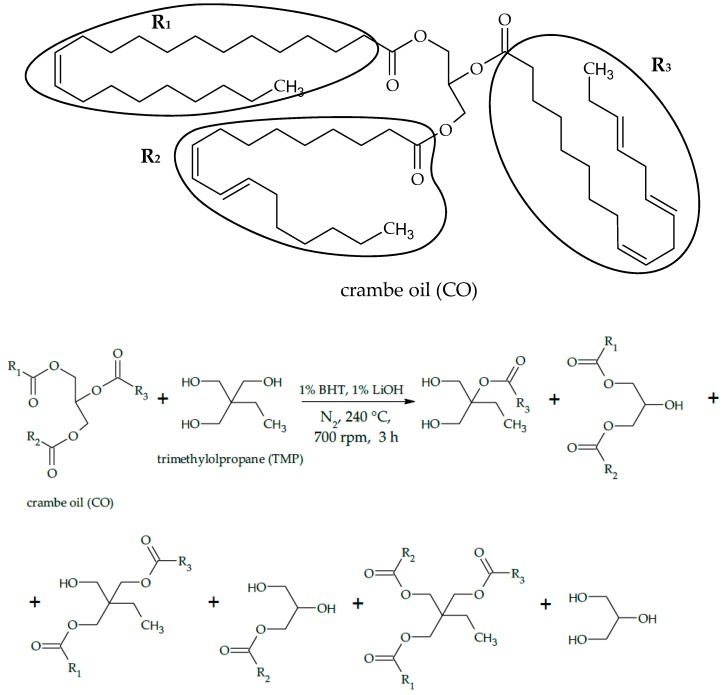
First step of modification of crambe oil.

**Scheme 3:**
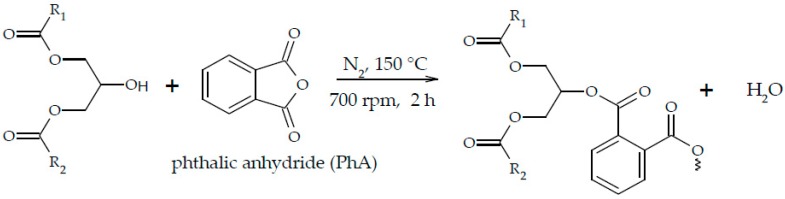
Reaction that occurs with all products formed in Scheme 2, especially with the –OH for polyester formation.

Polyester was produced by reacting a vegetable oil and oxygenated solvents based on literature with modifications [43,52,53]. Crambe oil (CO, 1995.2 g), trimethylolpropane (TMP, 804.6 g), butylated hydroxyl toluene (BHT, 1.0 g) and lithium hydroxide (LiOH, 0.50 g) under stirring and heating at 240 °C for 5 h in N_2_ atmosphere, and glycerin (a by-product) was removed during the reaction with Dean-Stark (Figure 5a and Scheme 2). The reaction progress was monitored by the methanol solubility test (3 mL methanol/1 mL polyester) and acidity index (mg KOH/g of polyester). When the polyester was totally solubilized in methanol and the acidity index was lower than 30, the reaction was completed, and the synthesis stopped. After 5 h of reaction, the temperature was lowered to 150 °C and phthalic anhydride (PhA, 888.6 g) was added under stirring and heating at 150 °C for 2 h in N_2_ atmosphere (Figure 5a and Scheme 3).

#### 3.2.2. Prepolymer Preparation

**Scheme 4:**
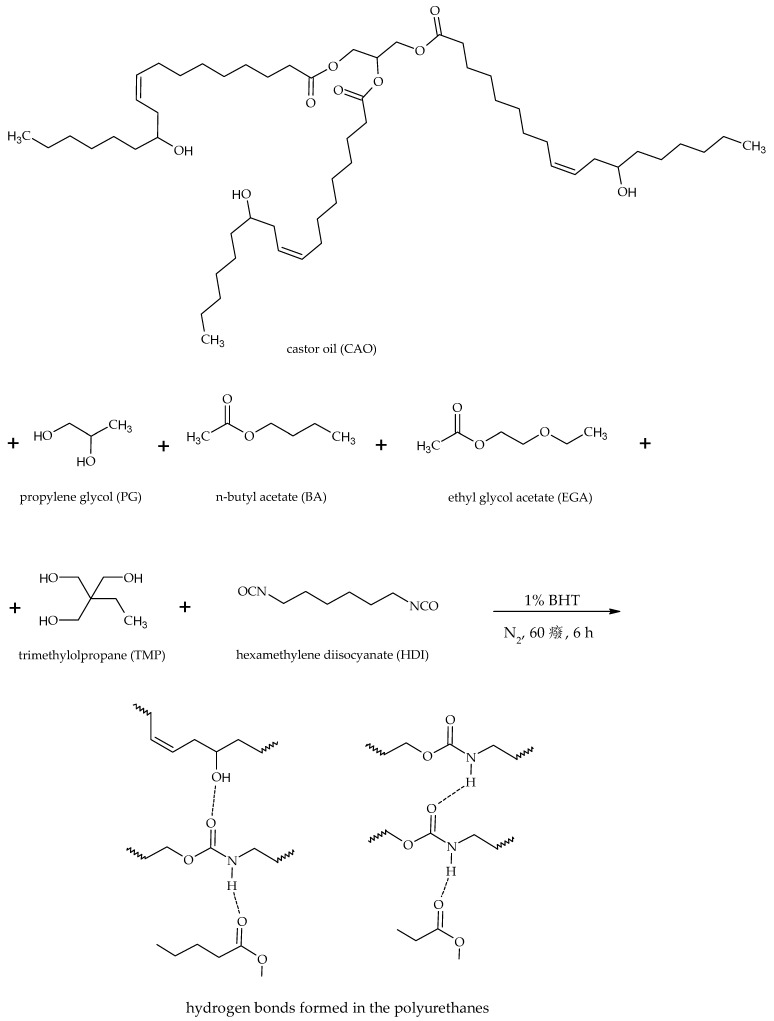
Prepolymer obtaining reaction.

Prepolymer was produced by reacting a vegetable oil and oxygenated solvents based on literature with modifications [43]. The prepolymer was prepared with castor oil (CAO, 250.6 g), trimethylolpropane (TMP, 93.86 g), butylated hydroxy toluene (BHT, 4.40 g), propylene glycol (PG, 24.34 g), n-butyl acetate (BA, 889.78 g), ethyl glycol acetate (EGA, 383.22 g), hexamethylene diisocyanate (HDI, 1046.16 g) under stirring and heating at 60 °C for 6 h in N_2_ atmosphere (Figure 5b and Scheme 4). 

#### 3.2.3. Characterization of Polyester and Prepolymer

##### Hydroxyl Groups, Solid Content, Solvent Content, and NCO Groups

Table 2 shows the physicochemical properties for the polyester (hydroxyl groups) and prepolymer (solid content and solvent content, NCO groups).

Crambe oil has no hydroxyl in its chemical structure, so the structure is modified by transesterification with trimethylolpropane (TMP). The hydroxyl groups (I_OH_) are all considered hydroxyls present in the chemical structure of the modified polyester. Thus, the result of the hydroxyl groups (151.6 mg KOH/g sample) confirms the modification of the raw material (crambe oil). According to the literature [26] the greater the quantity of free hydroxyls, the more the reaction medium is conducive to carrying out chain cross-linking reactions.

The prepolymer presented 56.1% solid and 43.8% solvent, being higher than values found in the literature [54,55], but like those discussed by Mannari and Massingill [56]. These values indicate a decrease in the amount of volatile organic compounds. The NCO groups is also an important feature for prepolymers, for example, for commercial application normally the NCO groups corresponds to a maximum of 20% [57] and this work achieved 6.26%. From this index, the required amount of hydrogen donor groups was calculated according to the NCO/OH molar ratio studied for each coating.

##### Attenuated Total Reflectance Fourier Transform Infrared Spectroscopy (FTIR-ATR)

**Figure 6:**
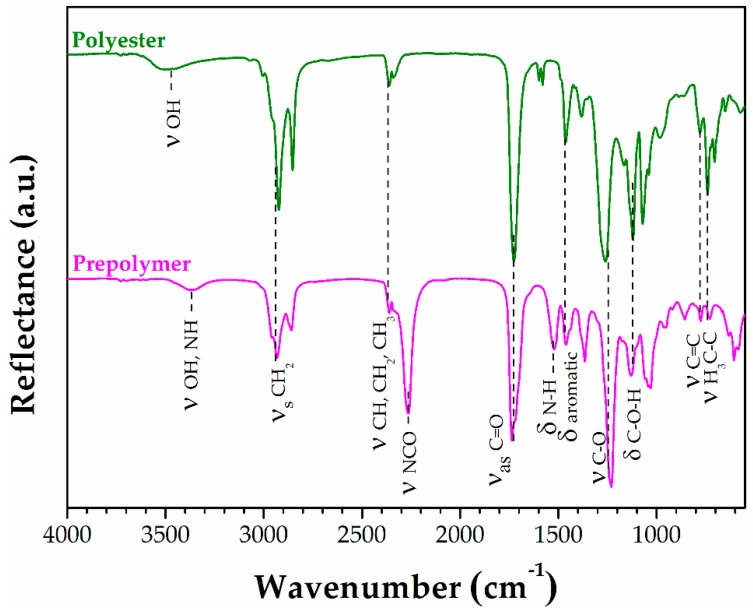
FTIR-ATR spectra for the polyester (**—**) and prepolymer (**—**).

Figure 6 illustrates the FTIR-ATR spectrum for polyester and prepolymer and the main attributions of the different bands in FTIR-ATR (normalized intensity of bands) stand out. 

The FTIR-ATR spectrum of polyester confirms the effectiveness of the crambe oil modification reaction (Scheme 2 and Scheme 3) by the presence of the –OH (3489 cm^−1^) band. The FTIR-ATR spectrum of prepolymer shows the presence of the characteristic NCO (2270 cm^−1^) band of free isocyanate groups in the chemical structure (Scheme 4) [58,59]. 

### 3.3. Preparation and Characterization of PU and PU-Modified Resins 

#### 3.3.1. Preparation of Polyurethane Resins

**Scheme 5:**
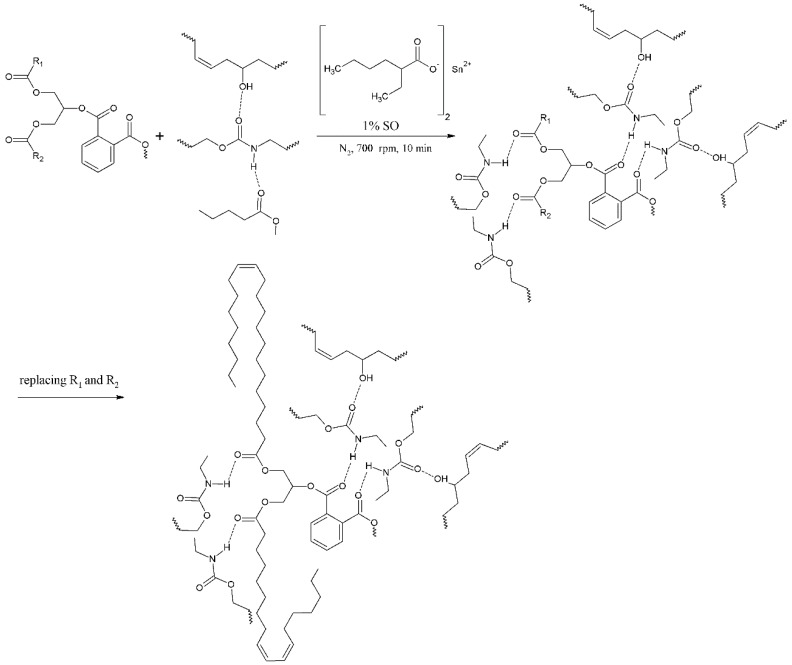
Preparation of PU-resin.

**Scheme 6:**
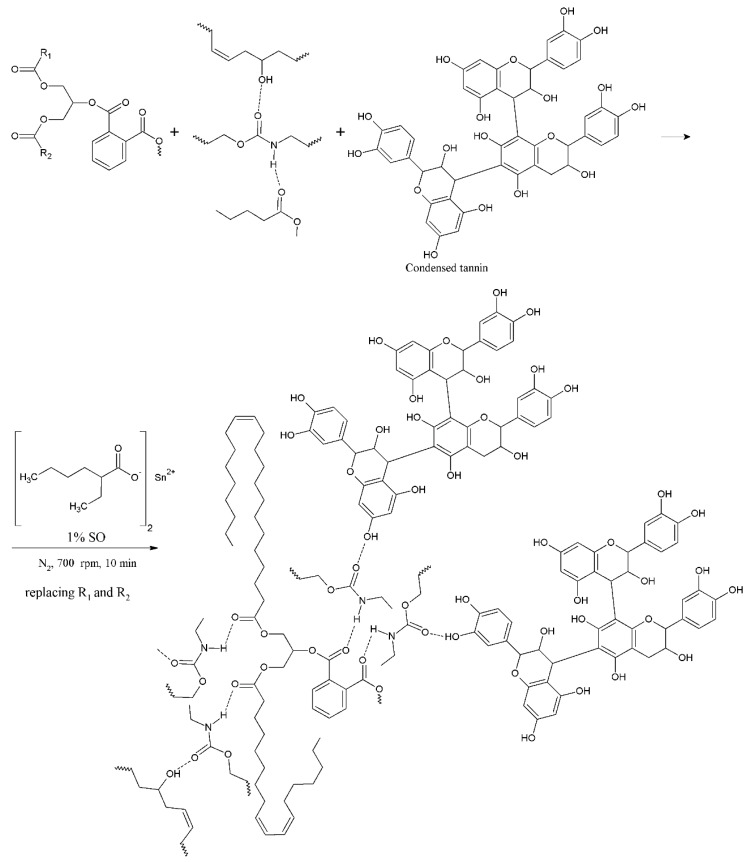
Preparation of PU-modified resin.

Polyester and prepolymer react in the presence of a catalyst, stannous octoate (SO, 1%), under stirring for 10 min to produce the PU resin (Figure 5c and Scheme 5). For the PU-modified resin, steps 1 and 2 were exactly the same as for the reference coating, except that condensed tannin was added (4.20 g) in the last step of the resin formulation (Figure 5c and Scheme 6). 

**Figure 7:**
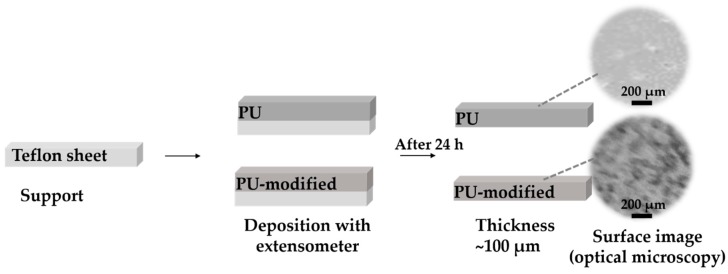
Scheme to obtain the monolithic resins of the PU and PU-modified with condensed tannin.

Figure 7 shows a scheme to obtain the monolithic resins. The Teflon sheet substrate was cleaned with deionized water and acetone and allowed to dry (~25 °C). The PU and PU-modified resins formulation was then cast using an applicator on the Teflon sheet substrate. The resin applied on the substrate was kept at room temperature (~25 °C) for 24 h to allow the solvent to evaporate.

#### 3.3.2. Characterization of PU and PU-Modified Resins

##### Attenuated Total Reflectance Fourier Transform Infrared Spectroscopy (FTIR-ATR)

**Figure 8:**
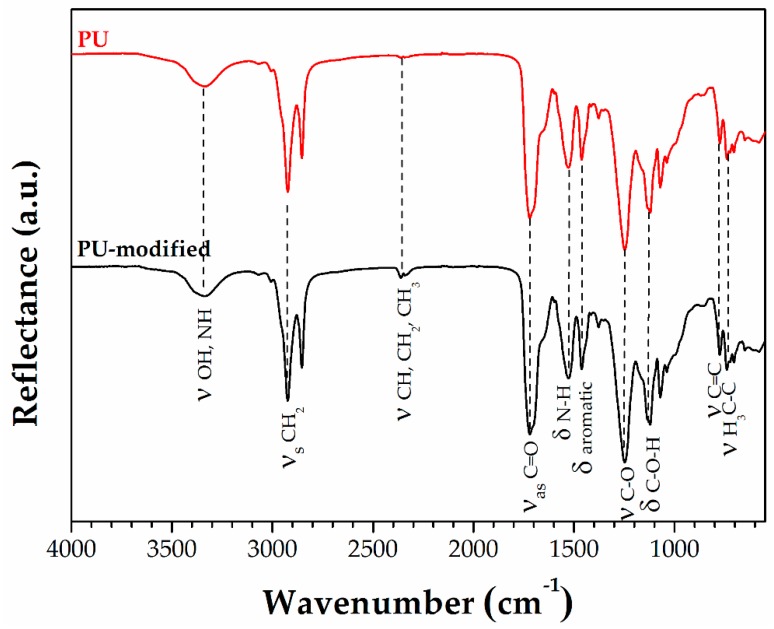
FTIR-ATR spectra for the PU resin (**—**) and PU-modified resin (**—**).

Figure 8 illustrates the FTIR-ATR spectrum for PU and PU-modified resins and the main attributions of the different bands in FTIR-ATR (normalized intensity of bands) stand out. 

In the FTIR-ATR spectrum of PU-resin, it was noted in the band attributed to NCO-free groups that some disappeared, indicating/confirming the complete reaction between the polyester and prepolymer (Scheme 5 and Scheme 6) [60]. These spectra are similar, but not the same, as presented by our group in previous studies [43], where the resin was applied on aluminum alloy, and in the present work the monoliths were analyzed. PU and PU-modified resins contain the characteristic bands of –OH (3346 cm^-1^), N–H (3340 and 1527 cm^−1^), C=O (1720 cm^−1^), C–O (1249 cm^−1^), O–H (1246 cm^−1^) and –O–H (1120 cm^−1^) bonds, which clearly show the formation of a urethane linkage [48].

##### Thermogravimetric Analysis (TGA)

**Figure 9:**
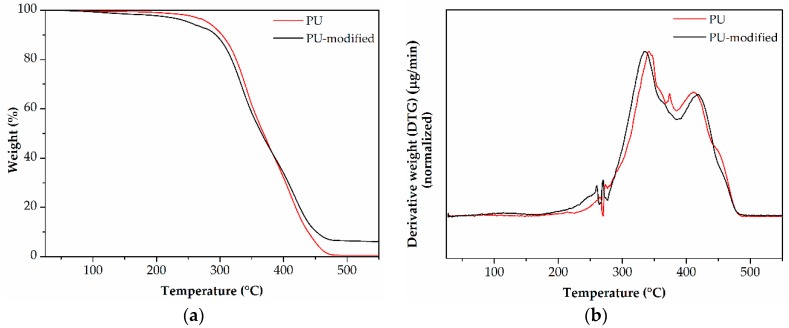
TGA weight loss (**a**) and derivatives of TGA data (**b**) of PU resin (**—**) and PU-modified resin (**—**).

Representative dynamic thermogravimetric analysis (TGA) results of PU and PU-modified resins are shown in Figure 9. Table 3 presents the attributions to the mass loss.

The PU and PU-modified resins exhibit no significant weight loss before 250 °C, indicating good thermal stability, as already observed for CO and CAO. Two not well-defined peaks are observed between ~260 and ~490 °C. The weight loss in the 260–350 °C range can be referred to the decomposition of chain extensors [61]; in the 360–490 °C interval, the thermal events can be associated with the breakup of prepolymer (rigid segment) of the polyester (flexible segment) [61] and 200–650 °C (two steps) can be referred to decomposition of polyurethane (PU) to form CO_2_ and water [62].

##### Raman Spectroscopy 

**Figure 10:**
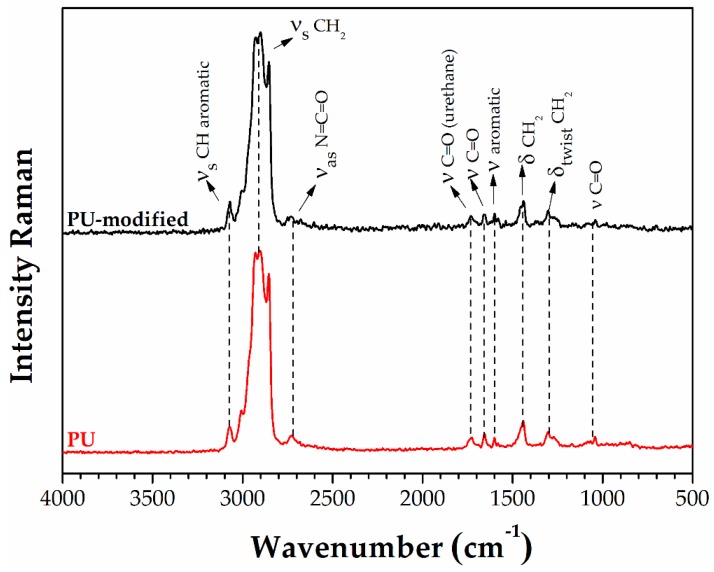
Raman spectra for the PU-blank and PU-modified (with intensity normalized).

Figure 10 shows the Raman spectrum for the resins (normalized for the intensities).

Note the presence of some more intense bands, which are characteristic of the formed polymer. The assignments of the bands were made according to previous studies on polyurethanes [63,64,65,66,67,68].

##### Artificial Defected Monoliths in Contact with Deionized Water

**Figure 11:**
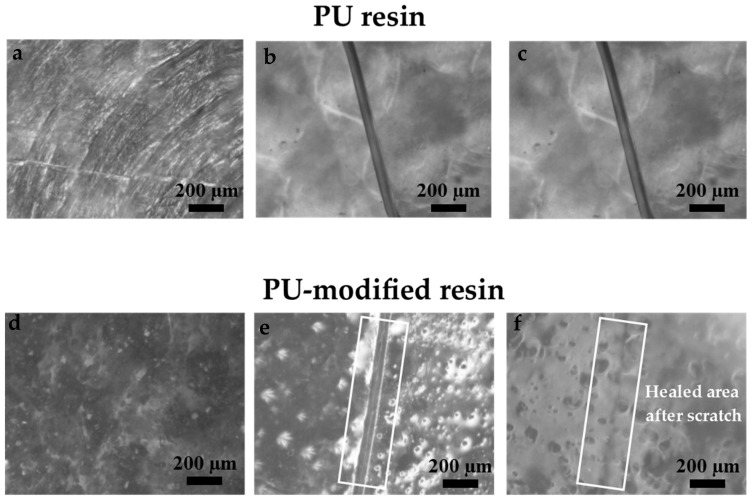
OM images of PU and condensed tannin-modified PU monoliths for the prepared surfaces (**a**,**d**) and after applying an artificial defect before (**b**,**e**) and after 4 h of immersion in deionized water (**c**,**f**).

Figure 11 shows the surface OM images of the PU and the condensed tannin-modified PU monolith obtained before and after immersion in deionized water when an artificial defect was made on the surface.

This figure clearly shows that for the PU the defect remained intact after immersion of the monolith in deionized water while for the condensed tannin-modified PU monolith the defect disappeared after some time in contact with deionized water. To demonstrate this process, videos (Figure S1) were recorded of both artificial-defected surfaces during the contact with deionized water for 4 h and it is clear that the defect remained almost unchanged for PU, while for the modified PU, the defect began to close by the movement of the coating walls limited by the defect until completely closing the defect after some immersion time. Other works have also studied the self-healing effect by microscopic images [59,69,70,71].

#### 3.3.3. Self-Healing Effect

**Figure 12:**
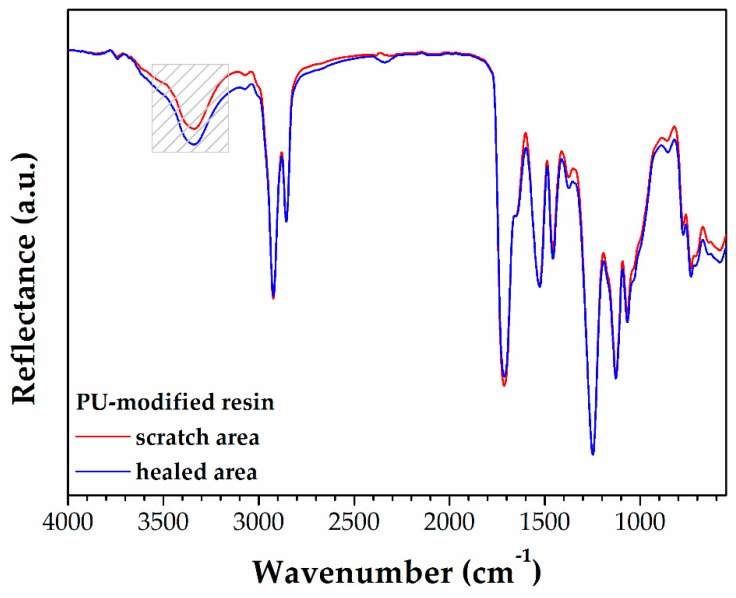
FTIR-ATR spectra of the modified-PU monolith with artificial defect and after having the defect repaired by immersion in water.

The above-described results (Figure 11 and Figure S1) allow us to propose that the incorporation of condensed tannin in the monolithic resin is responsible for the self-healing effect. The closing of the walls of the artificial defect in a condensed tannin-modified PU monolith was also observed when this type of coating was applied on AA2024 [43]. Tannin-modified PU monolith (100-μm thick) with a scratch of the 1000 μm long × 60 μm wide × 100 μm deep was automatically repaired when immersed in deionized water. When the modified resin was applied on AA2024, the healing effect was described based on hydrogen bond cleavage, then followed by the formation of a new hydrogen bond and confirmed by electrochemical localized techniques [43]. When tannin-modified PU was placed in contact with water, the swelling of the coating was insignificant, and the self-healing effect was observed, indicating that the self-healing mechanism can be the formation of hydrogen bonds. The FTIR-ATR (Figure 8) suggests an increase of the number of hydrogen bonds after adding condensed tannin to the PU resin. The FTIR-ATR spectrum of the tannin-modified PU monoliths with an artificial defect after having the defect repaired by immersion in water also indicates the increase of the intensity of the band attributed to hydrogen bond, demonstrating that the self-healing effect is based on hydrogen bond generation (Figure 12). 

**Figure 13:**
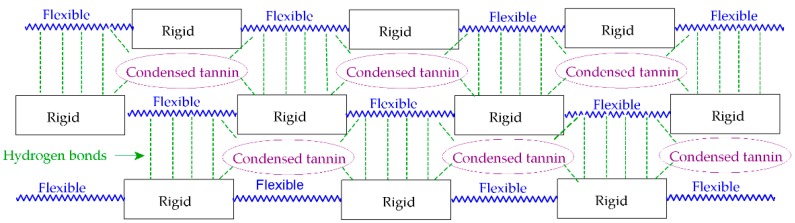
Scheme of hydrogen bonds between flexible and rigid segments of PU and hydrogen bonds between tanning and both segments.

It is very important to demonstrate that the self-healing property is a characteristic of the monolith, i.e., a property free of any metallic surface contribution. Therefore, the self-healing effect may be interpreted considering that the free OH groups present in tannin and some others created in the polyurethane by interacting with tannin when in contact with water or humidity allows the formation of new hydrogen bonds. Figure 13 schematizes the possibilities of hydrogen bonding formation between the flexible and rigid segments and condensed tannin. 

Therefore, the self-healing of the condensed tannin-modified PU coating is understood to function as follows: when an artificial defect or damage is produced on the coating surface, the humidity, water or aqueous solutions rapidly makes new hydrogen bonds—mainly with the tannin and the flexible segment of the polymer. This opens the possibility of hydrogen bond formation with the rigid segment of the polymer; therefore, the healing effect is based on hydrogen bond cleavage followed by new hydrogen bond formation. It is worth remembering that many of the properties of tannins are based on their ability to form hydrogen bonds [51,72]. Interactions involving the formation of new hydrogen bonds between a viscous system (here the polyester, prepolymer and nonmodified resin) in combination with a condensed tannin led to the formation of a flexible structure [1,73,74], mainly due to the greater proportion of polyester, more active than prepolymer, leading to a higher probability to form new hydrogen bonds with condensed tannin.

### 3.4. Applications of the PU and PU-Modified Resins

Special self-healing polyurethane coatings play an important role in the protection of aluminum alloys against corrosion in aggressive aqueous media, mainly in those containing chloride ions [21,22]. Polyurethane-based coatings were applied to protect AA1200 alloy and some formulation offered good barrier effect for 1 year of immersion in 3.5 wt % NaCl solution [22].

The resins synthesized in this work were applied on aluminum alloy AA2024-T3 for protecting it against corrosion and the self-healing effect investigated by electrochemical measurements in scratch zone [43]. The efficiency of the incorporation of tannin in the polyurethane resin (condensed tannin-modified PU) was already proved by FTIR-ATR and by the combination of localized electrochemical techniques with surface analysis (in the scratch and scratch-free zones). When immersion in NaCl 0.005 mol L^−1^ occurred, the self-healing effect start to act after 2–3 h and the coating was completely recovered at around 7 h of immersion in 3.5 wt % NaCl.

These results open a large window of applications of modified-PU resins in terms of their possible incorporation with other materials with different properties, such as corrosion inhibitors (encapsulated or not), reinforcing materials to increase the wear resistance, materials able to modify the hydrophobicity of the surface and to modify other properties of PU resins. As a consequence, modified-PU resins can be used to extend the lifetime of different metallic surfaces working in aqueous aggressive media or subjects, tribocorrosion conditions, as well as many other applications.

## 4. Conclusions

In this work, polyurethane (PU) produced from vegetable oils and condensed tannin-modified PU resin were successfully obtained and characterized by different chemical and physical techniques.

The formation of polyester through the modification of the crambe oil was confirmed by the determination of quantity of hydroxyl groups (151.6 mg KOH/g sample) and the prepolymer formation by the disappearance of the free NCO groups of hexamethylene diisocyanate. 

FTIR-ATR spectra confirmed that a condensed tannin was used. FTIR-ATR spectra were efficient in confirming the formation of polyester, prepolymer, PU and condensed tannin-modified PU resins, also reinforced by Raman spectra.

The good thermal stability of resins was confirmed by thermogravimetric analysis, since no appreciable weight loss below 250 °C was observed.

These studies open many possibilities for new vegetable oils-based polyurethane formulations and modified-polyurethane resins that can be used to extend the lifetime of different metallic surfaces, as well as the ability to work in aqueous aggressive media due to the generation of the self-healing property, or to better equip many other applications in materials science.

## Figures and Tables

**Table 1:** Results of compositional analysis profile of the methyl esters of crambe and castor oils.

Correspondent Fatty Acid	Chemical Name	Crambe	Castor
		(%)	
Palmitic acid	hexadecanoic acid	3.5	1.2
Stearic acid	octadecanoic acid	1.7	0.9
Oleic acid	*cis*-9-octadecenoic acid	15.1	3.6
Ricinoleic acid	12-hydroxy-9-*cis* octadecenoic acid	-	94.9
Linoleic acid	*cis*-,*cis*-9,12-octadecadienoic acid	7.4	6.2
Linolenic acid	*cis*,9,12,15-octadecatrienoic acid	5.1	0.7
Arachidic acid	eicosanoic acid	1.9	-
Gadoleic acid	*cis*,9-eicosenoic acid	6.9	0.5
-	11,14-Eicosadienoic acid	0.4	-
Behenic acid	docosanoic acid	3.6	-
Erucic acid	13-*cis*-docosenoic acid	52.9	-
DHA	*cis*-4,7,10,13,16,19-docosahexanoic acid	1.1	-
Lignoceric acid	tetracosanoic acid	5.2	-
-	*cis*-15-tetracenoic acid	2.3	-

**Table 2:** Physicochemical properties of polyester and prepolymer.

Properties			
Polyester		Prepolymer	
Hydroxyl groups (mg KOH/g sample)	151.6	**Solid content (%)**	56.1
		**Solvent content (%)**	43.8
		**NCO groups (%)**	6.26

**Table 3:** Attributions to the different thermal events.

Temperature (°C)	Weight Loss Due to:
260–350	Decomposition of active H sources (chain extenders)
360–490	Breakup of isocyanate (rigid segment) of the polyester (flexible segment)
200–650 (two steps)	Decomposition of polyurethane (PU)

## Supplementary Materials

The following are available online at https://www.mdpi.com/2073-4360/11/11/1890/s1, Figure S1 contains videos a and b.
