# Supplementary material for: Preparation of Polyurethane Monolithic Resins and Modification with a Condensed Tannin-Yielding Self-Healing Property
Source: Polymers (Basel). 2019 Nov 15;11(11):1890. doi: 10.3390/polym11111890 (PMC6918299; doi:10.3390/polym11111890)
Supplement: Supplementary file 1 [file polymers-11-01890-s001.zip › SM/polymers-624187 SM.docx]

Supplementary Material

Preparation of Polyurethane Monolithic Resins and Modification with a Condensed Tannin-Yielding Self-Healing Property

Jéssica Verger Nardeli ^1,^*, Cecílio Sadao Fugivara ^1^, Elaine Ruzgus Pereira Pinto ^2^, Wagner Luiz Polito ^2^, Younes Messaddeq ^2^, Sidney José Lima Ribeiro ^2^ and Assis Vicente Benedetti ^1,^*

^1^ São Paulo State University (UNESP), Institute of Chemistry, Department of Physical-chemistry, PO Box 355, Araraquara, 14801-970, SP, Brazil

^2^ São Paulo State University (UNESP), Institute of Chemistry, Department of General and Inorganic Chemistry, PO Box 355, Araraquara, 14801-970, SP, Brazil

***** Correspondence: jeh.nardeli@gmail.com (J.V.N.), assis.v.benedetti@unesp.br (A.V.B.)

video-a

video-b

**Figure S1**. Videos of PU (**a**) and condensed tannin-modified PU (**b**) monoliths after applying an artificial defect during all immersion time in deionized water.
